# Retraction: Human Epididymis Protein-4 (HE-4): A Novel Cross-Class Protease Inhibitor

**DOI:** 10.1371/journal.pone.0305589

**Published:** 2024-06-11

**Authors:** The PLOS ONE Editors

Following the publication of this article [1], concerns were raised regarding images in Figures 1 and 7. Specifically,

- In Figure 1D, the 45kDa marker in lane I appears similar to the band in lane III, and the 25kDa and 18.8kDa markers in lane I appear similar to each other.
- In Figure 7A, the following areas appear similar despite representing different experimental conditions:
  - 35, 25, 18, and 14 kDa markers in lane 1.
  - HE-4 bands in lanes 2 and 3.
  - Papain bands in Lanes 4 and 5.
  - HE-4 bands in lanes 6–9.
- In Figure 7B, all the markers in lane 1, the 35kDa band in lane 9, and the areas surrounding these bands appear more similar than expected.

In response to the above concerns, the corresponding author acknowledged that there are similarities but refuted that the indicated areas are identical. Images and alternative experimental results provided in support of these figures did not resolve the concerns.

In light of the concerns affecting multiple figure panels that question the integrity of these data, the *PLOS ONE* Editors retract this article.

NC, AKT, and SY did not agree with the retraction and stand by the article’s findings. MS, SD, and SS either did not respond directly or could not be reached.
